# Predictors of non-adherence to medication and time to default from treatment on HIV infected patients under HAART: a comparison of joint and separate models

**DOI:** 10.4314/ahs.v22i1.53

**Published:** 2022-03

**Authors:** Koyachew Bitew Abebe, Awoke Seyoum Tegegne

**Affiliations:** 1 Department of Statistics, Debre Tabor University Debre Tabor, Ethiopia. koyeabebe@gmail.com; Cell: +251974450237; 2 Department of Statistics, Bahir Dar University Bahir Dar, Ethiopia. bisrategebrail@yahoo.com; Cell: +251918779451

**Keywords:** Non-adherence, separate model, joint model, time to default, HAART

## Abstract

**Background:**

Ethiopia is one of the Sub-Saharan Africa with the highest number of people living with HIV. Amhara region is one of the regions in the country in which many people are under medication. The main objective of this research was to identify significant predictors of non-adherence to medication and time to default from treatment for HIV infected patients under HAART.

**Methods:**

A retrospective secondary data were obtained from a random sample of 220 HIV patients under HAART. Separate and joint modeling approaches were conducted in data analysis. Joint modeling was conducted for analysis of non-adherence to medication and the time to default from treatment. In the joint model, a GLMM and Cox PH sub-models were fit together for non-adherence to medication and time to default from treatment.

**Results:**

The significant predictors for the variables of interests in current investigation were length of visiting time(AOR of 95% CI=0.866 (0.752, 0.997), female patients(AOR of 95% CI= 0.219 (0.067, 0.717)), patients disclosed the disease(AOR of 95% CI= 0.353 (0.194,0.641)), patients who got social support(AOR of 95% CI= 0.252 (0.194,0.631)), patients living with parter(AOR of 95% CI=0.188 (0.042,0.844)), patients with owner of cell phone(AOR of 95%CI= 0.272 (0.081,0.916)), urban HIV patients(AOR of 94%CI= 0.238 (0.078,0.722)), patients with working functional status(AOR of 95% CI= 0.234 (0.079,0.692)), patients with normal BMI(AOR of 95% CI=0.921 (0.881, 0.963)), patients with high baseline CD4 cell count(AOR of 95% CI=0.873 (0.552, 0.997)).

**Conclusion:**

Some groups of HIV patients were non-adherent to medication and defaulted from treatment. Health related education is recommended for non-adherent patients to be adherent for the prescribed medication and live long in the treatment.

## Introduction

Human immunodeficiency virus (HIV) slowly destroys the immune system, which is our body's ordinary defense against illness^1^. AIDS is a chronic and communicable disease caused by HIV^2^. The last stage for HIV is AIDS and it is the time that the body of patients can no longer defend itself from disease. According to UNAIDS report, about 36.7 million people were living with HIV globally in 2018 3.

Ethiopia is one of the few African countries with the highest number of people under HAART because of HIV^4^. Currently, the distribution of HIV and people under HAART in the Amhara region including the catchment area of Felege-Hiwot Teaching and Specialized Hospital (North-west Ethiopia) is very high5. Because of its large population size in the country, large number of people living with the virus are under treatment^5^, ^6^. Some people under treatment show significant improvement on their health status but not true for the others because of their non-adherence to medication and such non-adherence to medication patients became defaulters from treatment^7^, ^8^.

Being non-adherence to medication and default from treatment may result in regimen failure, immune suppression, emergence of resistance of viral strains and higher treatment costs^9^, ^10^. Hence, high levels of follow ups are necessary for consistent viral suppression^11^ and for avoidance of resistance^12^. Non-adherence to medication causes treatment and virological failures, recurrent development of opportunistic infections and ultimately deaths among people living with HIV under HAART^13^–^15^. As a result, non-adherent patients are at high risk of illness and death because of AIDS related conditions^16^.

It is usually suggested to model repetitive records and time-to-event data jointly using shared random effects, considering the relation between repeated longitudinal and time to event data on the same individual^17^. Recently, predictors of adherence and time to default researches are conducted separately; however, such studies did no investigate the common predictors of these responses and the association between the two. Many of the studies are cross sectional and did not investigate the repeated observations on the same subject. Therefore, the main objective of current investigation was to identify significant predictors for non-adherence to medication and time to default from treatment for HIV infected patients under HAART at Felege Hiwot Teaching and Specialized Hospital (North Western Ethiopia). As far as authors' knowledge is concerned, there is scarcity of literatures conducted jointly and measured repeatedly for HIV positive people. This made current investigation essential to assess whether the international experience also work in the study area.

## Methods

### Study Area and design

A retrospectively cohort study design was conducted on 220 HIV infected patients under HAART at Felege Hiwot Teaching and Specialized Hospital, North-Western Ethiopia. The hospital is serving for all HIV patients, receiving treatment with full regional laboratory equipment.

### Source of data

The data was secondary that has been collected from participants by the health staff for treatment purpose. The data were therefore, recorded in each patient's card and documented in ART section of the hospital. A joint model was used in predicting the two response variables.

### Sample size and Sampling procedures

A random samples of 220 samples were selected considering their ART identification number. Cochran's formula was conducted in determining sample size^18^, ^19^. The samples were selected using simple random sampling technique using their card numbers in the hospital.

### Inclusion criteria

HIV infected patients under HAART with at least two repeated measures of non-adherence to medication performance and whose follow-ups were from January, 2014 up to December, 2017.were included in this investigation.

### Variables under current investigation

The dependent variables for this study were non-adherence to medication and time to default from treatment. The predictor variables were sex, age category, marital status, level of education, social support, social violence, residence area, existence of mental depression, religion, functional status, occupational status, opportunistic infectious disease, cell phone ownership, WHO stages of HIV, level of disclosure of the disease, BMI category, baseline CD4 cell count and baseline hemoglobin. The categories of each predictor are indicated in Table 1.

**Table 1:** Description of potential predictor variables for variable of interests (n=220)

Characteristics	Category	Frequency	Percentage
sex	Female	136	61.80
	Male	84	38.20
Marital status	Without partner	106	48.18
	With partner	114	51.82
Age category	Children (age <15 years)	112	50.9
	Adults(>= 15 years)	108	49.1
Level of education	No education	69	31.36
	primary	55	25.00
	Secondary	56	25.45
	Tertiary	40	18.19
Social support	No	109	49.55
	Yes	111	50.45
Existence of mental depression	No	115	52.3
	Yes	105	47.7
Residence	Rural	111	55.00
	Urban	99	45.00
Religion	Orthodox	110	50.00
	Muslim	94	42.73
	Others	16	7.27
Functional status	Ambulatory	13	5.90
	Bedridden	22	10.00
	Working	185	84.10
Occupation status	Unemployed	65	29.55
	Employed	92	41.82
	Others	63	28.63
Opportunistic infectious disease	No	133	60.45
	Yes	87	39.1
Owner of cell phone	No	86	39.1
	Yes	134	60.90
WHO stages	Stage I	59	26.37
	Stage II	58	31.36
	Stage III	69	26.37
	Stage Iv	34	15.45
Disclosure of the disease	No	62	28.18
	Yes	158	71.82
BMI	underweight	71	32.27
	Normal	112	50.91
	Overweight	37	16.82
Average baseline CD4/mm3		211	
Average Baseline HGB/gl		9.46	
Length of observational time/visiting time	full observational time defaulted from treatment	16visits	84.1% 15.9%
Adherence level	Non-adherence to medication		46%
	Adherent to medication		54%

### Self-reported variables

Variable like number of pills not taken, dietary instruction, the time when pills were taken, existence of mental depression, existence of social violence by people living together, existence of social support, existence of medication allergic at initial time were reported by participants and recorded carefully in each patient's chart.

Measures of non-adherence and time to default: Non-adherence to medication was measured using pills counts conducted by health staff at the treatment site. Patients were directed to bring all medication bottles and unused pills at each treatment visit but patients were not aware that unused pills used for auditing and calculation of non-adherence level. Hence, non-adherence = *100%19. In this regard, a patient was categorized as non-adherence to medication if he/she adhered less than 95% of the prescribed medication by the health staff.

In current investigation, defaulters were patients those did not come back to the ART clinic until the end of study period (31st December, 2017). A defaulter could be existed as a result of death, transferring to other hospital and loss-to follow up. Therefore, being defaulter from treatment was variable of interest for the survival analysis^16^.

At the initial time of the treatment, the patients were directed to visit the hospital monthly for the first six months and quarterly for the remaining study period. Hence, there were 16 follow ups for those patients with full length of observation in the study period. The reason for monthly follow ups at initial time was to follow up whether there existed medication side effects like mental depression, skin scratch and any other medication allergic on individuals at the initial time.

### Data Analysis

R version 3.4.4 statistical software was used to analyze the data. A Binary logistic regression was employed for longitudinal outcome variable (non-adherence to medication) and Cox proportional hazards (PH) model was also employed for analysis of time to default from treatment. Statistical decision was made at 5% level of significance. Both separate and joint modeling approaches were conducted for modeling the longitudinal non-adherence to medication and time to default data. In joint modeling procedures a set of random effects and the correlation between the two outcomes were considered.

### Formulation of joint models

A direct formulation of joint modeling for both response variables with the introduction of Placket latent variable assumption in modeling longitudinal and survival outcomes was used in current investigation^20^. To obtain valid inferences, the joint model could account for the correction among the outcomes and effects of different factors. The joint generalized linear mixed model assumes that each outcome and the univariate models are combined throughpecification of joint multivariate distribution for all random effects. Furthermore, the mixed model can be applied with specification of marginal distribution, which is conditional on correlated random effect. To assess the association between non-adherence to medication and time to default, the joint generalized linear mixed model was fitted. In this model, the correlation between the two responses was specified through the random effect structure (alpha) assuming separate random intercept for each outcome and combining them by imposing joint multivariate distribution on the random intercept. Method of parameter estimation for joint model was conducted for the subject-specific and marginal residuals.

## Results

### Descriptive Data Analysis

The baseline socio-demographic and clinical characteristics of patients included in the analysis are presented in Table 1. Table 1 indicates that 61.8% of patients were female, 50.9% of the participants were children (whose age < 15 years), 51.82% were living with a partner: 31.4 % of patients had no education; 55% of them were rural residents; half percent of the patients were orthodox followers, 84.1% of the patients were with working functional status (i.e., an individual able to perform the usual work).

Table 1 also indicates that about 60.5 % had no past opportunistic infections, 42% of the patients had cell phones, 31.4% of patients were classified as WHO stage III; 71.8% disclosed their HIV disease status, 50.9% of them were categorized under well nourished, 41.8% of them are employed and around 50% of the patients got social support. At enrolment, the average baseline CD4 cell count per mm^3^ and baseline hemoglobin per gl, were 211 and 9.46 respectively. About 46 % of the patients were non-adherence to medication, 15.9% of the patients were defaulted from treatment and 84.1% of them were (censored) at the end of study period.

The Log-rank test was used for the difference estimated survival time among the categories of predictors and the result revealed that there was a statistically significant difference of survival time among the categories of variables.

Since patients visited the hospital with unequal time interval, unstructured was the appropriate covariance structure relative to other covariance structures and this was also supported by the AIC and BIC comparisons, considering the smallest is the best^21^. In data analysis, first univariate data analysis was performed and next multivariable model was fitted at 5% level of significance^22^.

The Binary logistic regression model was conducted for the analysis of the separate model of non-adherence to medication as shown in Table 2. Similarly, the Survival Cox PH model was fitted for time to default from treatment as indicated in Table 3.

**Table 2:** Predictors of non-adherence to medication in panel data analysis

Parameters	Estimates	SE	AOR (95% CI)	p-value
Intercept	4.90	1.6300	134.7 (5.910, 304.97)	0.0027*
Length of observation	-0.14	.0800	0.87(0.741,0.902)	0.0150*
time/visits				
Sex (Ref.=male)				
female	-1.52	0.6000	0.220 (0.071,0.723)	0.0121*
Age category(Ref.= children)	-0.323	0.4532	0.724(0.453, 0.924)	0.006*
adults				
Religion (Ref.=others)				
Muslim	0.0613 0.4800	0.0271 0.2151	1.063 (0.08, 1.121)	0.237
Orthodox			1.616 (0.059, 2.465)	0.571
Disease disclosure				
status(Ref.=no)	-1.0413	0.3046	0.353 (0.194,0.641)	0.0006*
Yes				
Social Support(Ref.=No)				
Yes	-1.235	0.865	0.291(0.082, 0.423)	0.0043*
Marital status(Ref.= without partner)	-1.6692	0.7654	0.188 (0.042,0.844)	0.0291*
With partner				
Residence (Ref. =Rural)				
Urban	-1.4369	0.5669	0.238 (0.078,0.722)	0.0112*
Existence of mental depression(Ref.= No)	0.642	0.654	1.900(1.043, 2.625)	0.0121*
Yes				
Opportunistic infectious disease(Ref.=No)	0.463	0.268	1.5888(1.232,	0.0052*
Yes			2.352)	
Existence of social violence (Ref.= No)	0.542	0.654	1.719(1.021, 2.182)	0.0022*
Yes				
Ownership of cell phone (Ref. = No)	-1.300	0.6185	0.273 (0.081,0.916)	0.0356*
Yes				
WHO stages (Ref. =stage 4)	-0.2387	0.2529	0.788 (0.479,1.293)	0.3450
Stage 3	-0.9064	0.4040	0.404 (0.183,1.892)	0.2492
Stage 2	-1.4385	0.6330	0.237 (0.067,1.821)	0.0631
Stage 1				
Level of education(Ref.= Tertiary)	-0.1287	0.6429	0.879 (0.479,1.293)	0.6450
Secondary	-0.9064	0.8240	0.404(0.183,1.892)	0.6392
Primary	-1.4385	0.7230	0.237 (0.067,1.828)	0.0831
No education				
Functional status (Ref. =Ambulatory)	1.4360	0.7222	4.203	0.0469*
Bedridden	-1.4508	0.5554	(1.021,17.314)	0.0090*
Working			0.234 (0.079,0.696)	

* stands for statistically significant variables at p < 0.05

**Table 3:** Cox Proportional Hazard models for time to default from treatment

Parameters	Estimates	SE	HR (95% CI)	p-value
Sex(Ref.=male)				
female	-1.7793	0.6619	0.169 (0.046, 0.618)	0.0072*
Age category(Ref. =children)	0.642	0.654	1.900(1.043, 2.352)	0.0121*
Adults				
Functional Status (Ref. =Ambulatory)	1.4330	0.6622	4.191(1.145, 15.347)	0.0305*
Bedridden	-2.2521	0.6654	0.105 (0.029, 0.388)	0.0007*
Working				
Disclosure(ref.=no)				
Yes	-1.0517	0.3766	0.349 (0.167, 0.731)	0.0052*
Social Support(Ref.=No)				
Yes	-1.235	0.865	0.291(0.082, 0.423)	0.0043*
Opportunistic infectious disease(Ref.=No)	0.642	0.654	1.900(1.043, 2.352)	0.0021*
Yes				
Existence of mental dep.(Ref.= No)	0.642	0.654	1.900(1.043, 2.352)	0.0121*
Yes				
Exist. of social violence (Ref.=No)	0.542	0.654	1.719(1.021, 2.182)	0.0022*
Yes				
Ownership of cell phone (Ref. = No)	-1.300	0.6185	0.273 (0.081,0.916)	0.0356*
Yes				
Baseline HGB	-1.8900	0.6260	0.151 (0.044,0.515)	0.0025*
Baseline CD4 cell	-0.0041	0.0015	0.996 (0.993,0.999)	0.0064*
count				
WHO stages (Ref. =stage 4)	-1.0771	0.5054	0.341(0.126,0.917)	0.0331*
Stage 3	-1.2391	0.5169	0.289 (0.105,0.798)	0.0165*
Stage 2	-2.2309	0.6615	0.107 (0.029,0.393)	0.0007*
Stage 1				
BMI category(Ref. =obesity)	-0.9520	0.3930	0.386 (0.179,0.834)	0.0154*
Normal	1.4360	0.7222	4.204 (1.021,17.313)	0.0469*
Underweight				

* stands for statistically significant variables at p < 0.05

In Table 2, length of observation time, female patients, patients disclosed their disease status, patients living with partners, urban patients, patient who owned cell phone, patients with working functional status, patients who got social support from people around them, patients who did not have mental depression, adult patients and patients at normal BMI were negatively associated with the level of non-adherence to medication in this study. But, bedridden and ambulatory functional status, non-educated patients and malnourished patients were positively associated with the variable of interest.

In Table 3, female patients, patients who had working functional status, patients disclosed their disease, high baseline CD4 cell count, high baseline hemoglobin, patients who got social support, patients with ownership of cell phone and patients at normal BMI were negatively associated with the hazard of defaulting times from treatment. Hence, such variables significantly affect the study subject for long waiting time/large number of follow ups in the study period^22^.

The joint modeling of the two response variables name- ly non-adherence to medication and time to default was also conducted as shown in Table 4.

**Table 4:** Joint predictors of non-adherence to medication and time to default

Parameters	Estimates	Std. Error	AOR (95% CI)	p-value
Intercept	0.9026	1.6328	2.466 (1.487, 4.996)	0.0027*
Length of observation time/visits	-0.1442	0.0721	0.866 (0.752, 0.997)	0.0455*
Baseline CD4 cell count	-0.1351	0.0721	0.873 (0.552, 0.997)	0.0455*
Baseline HGB	-0.8900	0.6260	0.4106 (0.044, 0.515)	0.0025*
Sex (Ref.= male)				
female	-1.5186	0.6049	0.219 (0.067, 0.717)	0.0121*
Age category(Ref. =Children)	-0.0453	0.231	0.9557(0.7688, 0.9987)	0.0011*
adults				
Religion (Ref.=others)				
Muslim	0.0613 0.4801	0.0270 0.214	1.063 (0.008,1.121)	0.2320
Orthodox		9	1.616 (0.059,2.465)	0.2551
Disclosure(Ref.=no)				
Yes	-1.0413	0.3045	0.353 (0.194,0.641)	0.0006*
Exist. of mental dep.(Ref.=No)	0.0613	0.846	1.063(1.002, 2.063)	0.0016)*
Yes				
Social support(Ref.=No				
Yes	-1.0413	0.3045	0.252 (0.194,0.631)	0.0026*
Marital status(Ref.=without partner)				
With partner	-1.6691	0.7653	0.188 (0.042,0.844)	0.0290*
Residence (Ref. = Rural)				
Urban	-1.4370	0.5665	0.238 (0.078,0.722)	0.0112*
Ownership of cell phone (ref. = No)				
Yes	-1.3010	0.6182	0.272 (0.081,0.916)	0.0353*
WHO stages (ref. =stage 4)				
Stage 3	0.5387	0.2525 0.404	1.714 (1.356,1.845)	0.0329*
Stage 2	0.2064	0	1.229 (1.184,1.453)	0.0249*
Stage 1	0.1852	0.6330	1.204 (1.067,1.523)	0.0231*
education(ref.=tertiary)				
no education	0.5800	0.5650	1.786 (1.104, 3.693)	0.0048*
primary	0.4900	0.5620 0.461	1.632 (1.10, 3.453)	0.0081*
secondary	0.0849	1	1.088 (1.009,2.306)	0.0186*
Functional status (ref. =Ambulatory)				
Bedridden	0.4361	0.7220	1.547 (1.021,3.309)	0.0466*
Working	-1.4509	0.5552	0.234 (0.079,0.692)	0.0086*
BMI category(Ref. =obesity)	-0.0821	0.0225	0.921 (0.881, 0.963)	0.0003*
Normal	0.0613	0.0271	1.063 (0.008, 1.121)	0.2360
Underweight				
Association parameter()	0.1339	0.3741	1.143(1.004, 1.432)	0.0258*

* stands for statistically significant variables at p < 0.05

Table 4 indicates that as number of follow ups increased by one unit, the odds of being non-adherence to medication was decreased by 13.4% for ART treatment [AOR= 0.866; 95% CI: (0.753, 0.995), p-value=0.0455] given that the other variables constant.

The odds of being non-adherence to medication for female patients was decreased by 78% as compared to male patients given that the other variables constant [AOR= 0.219, 95% CI: (0.067, 0.717), p-value =0.0221]. The odds of being non-adherence to medication for patients disclosed the disease to people living together was decreased by 64.7% as compared to those patients who did not disclose their disease [AOR=0.353, 95% CI; (0.194, 0.641, p-value=0.0006] given the other predictors constant. The odds of being non-adherence to medication for adult patients was decreased by 4.6% as compared to children [AOR=0.956, 95% CI; (0.768, 0.999), p-value = 0.0011] given the other predictors constant.

Existence of mental depression has significant effect for the patient to be non-adherent. Hence, the odds of being non-adherence to medication for mentally depressed patients was increased by 6.3% as compared to those mentally non-depressed patients [AOR=1.063, 95% CI; (1.002, 2.063), p-value = 0.0016], considering the other variables constant. The odds of being non-adherence to medication for patients who got social supports was decreased by 74.8% given the other variables constant [AOR= 0.252, 95% CI; (0.194, 0.631), p-value = 0.0026]. The odds of being non-adherence to medication for a patient living with partner was decreased by 81.2% as compared to those patients living without partner, keeping the other variables constant [AOR= 0.188, 95% CI: 0.042, 0.844, p-value = 0.0290]. Similarly, the odds of being non-adherence to medication for patients who owned cell phone was decreased by 72.8% as compared to those patients living without cell phone, keeping the other variables constant[AOR= 0.272; 95% CI: (0.081, 0.916), p-value = 0.0353].

The odds of being non-adherence to medication for urban patients was decreased by 76.2% as compared to rural patients [AOR= 0.238, 95% CI; (0.078, 0.722), p-value=0.0112] given the other variables constant.

Level of education also played a significant role in predicting the variable of interest. Hence, the odds of being non-adherence to medication for uneducated patients was increased by 78.6% as compared to tertiary educated patients given the other predictors constant[ AOR=1.786, 95% CI; (1.104, 3.693), p-value=0.0048]. The odds of being non-adherence to medication for patients with working functional status was decreased by 76.6% as compared to patients with ambulatory given the other variables constant[ AOR=0.234, 95% CI; (0.079, 0.692), p-value = 0.0086]. Patients' BMI also had significant role for the variation of non-adherence to medication, Hence, the odds of being non-adherence to medication for normal BMI patients was decreased by 7.9% as compared those patients with abnormal BMI (underweight or obesity patients) given the other variables constant[AOR=0.921, 95% CI; (0.881, 0.963), p-value = 0.0003].

In current investigation, as the number of CD4 cell count increased by one cell/ mm3, the odds of being non-adherence to medication was decreased by 16.7% given the other variables constant[ AOR=0.873, 95% CI ;(0.552, 0.997), p-value=0.0455]. For a unit increase of baseline hemoglobin, the odds of being non-adherence to medication was decreased by 39% given the other factors constant [AOR=0.4106, 95% CI; (0.044, 0.515), p-value=0.0025].

WHO stages also had a significant role for prediction of non-adherence to medication. Hence, the odds of being non-adherence to medication for patients with WHO stage3 was increased by 71.4% as compared to patients with WHO stage4 (AOR=1.714, 95% CI: (1.356, 1.845), p-value=0.0329). Similarly, the odds of being non-adherence to medication for patients with WHO stage2 was increased by 22.9% as compared to patients WHO stage4 (AOR=1.229, 95% CI: (1.184, 1.453), p-value=0.0249) given the other conditions constant.

### Comparison of Separate and Joint Model

Separate and joint model were compared based on the standard errors computed in parameter estimations. It is known that, the smaller standard error is associated with the model fits the data better. Hence, joint model had narrow confidence interval which indicates its standard error is smaller for all significant predictors as compared to separate model(16). As indicated in Table4, the estimates of the association between parameters under joint model were significantly different from zero, this indicates that two outcomes were correlated to each other and this helped us to make valid inferences and conclusions. In this way, joint model provided more accurate estimates and more efficient inferences than separate models. This method of estimate enables investigators to make efficient conclusion about study population and recognize effects of the variables after correctly controlling the interaction among these processes.

The statistical significance of the association parameter (Alpha) is also evidence that the joint model was better than the separate models. The positive sign for association parameter (Alpha) indicates that the two outcomes namely; non-adherence to medication and time to default from treatment were positively correlated.

## Discussion

The most repeated responses obtained from the HIV patients and most sited reason to be non-adherence to medication are forgetfulness. As it is stated in other studies, forgetfulness has being linked to HIV-associated neurocognitive disorders and consequently to non-adherence to medication^20^, ^21^

Male HIV patients are more non-adherent to medication and have short period of time to be defaulted from treatment as compared to females. The possible reason for this might be that females had good experience in taking pills for family planning (birth control) and this leads to have less probability of being non-adherent to medication as compared to males. The other possible reason for such differences is males in the study area are exposed for smoking and alcohol consumptions and patients after drinking alcohol may not take medication because of the toxic interaction of the two. Hence, females have series follow-ups for treatment as compared to males which is consistent with previous investigations^23^, ^24^. One of the other previous studies, contradicted with current result, states that females are so busy in take care of their children and they forgot to take pills on time as compared to males and this leads to be more non-adherent to medication^25^.

The main reason for non-adherence to medication is forgetfulness and cell phone plays significant effect for the patients to be adherent. Hence, patients can use their cell phone as reminder/memory aid to take pills on time and this leads them to be adherent to medication. The alarm of cell phone also helps to be programed to visit the hospital on the date prescribed by health staff. This finding is consistent with other study^26^.

Patients disclosed their disease status to their families living together can be encouraged by their partner to take pills on time and to take treatment properly as well as they give social support and this leads for the patient being strict on medication and follow ups of treatments. Patients who disclosed the disease might make them picking up their medicine on time without frustration and due to this, defaulting from treatment may be lower. It is possible to say that, due to fear of stigmatization, patients who did not disclose the disease would choose to skip their medication when in public and away from home. The hazard of defaulting for patients disclosed their disease status is lower than patients who didn't disclose the disease. This result is supported by one of the previous studies^26^ and contradicted by another study^27^. The argument from the study with result opposed to current result is that patients disclosed the disease got social violence from the community^25^. Hence, this result needs further investigation.

Urban HIV patients have better access of health institutions that can give HIV treatment services as compared to rural patients. Long distance from their residence, for rural patients also makes difficulties for close follow ups and being non-adherence to medication. This leads for rural patients to become more non-adherence to medication as compared to urban patients^28^. Rural patients visit the health institution not based on the date given by health staff rather they visit whenever they are free from regular work^29^.

Patients living with partner have better adherence performance as compared to those living without partners. The potential reason for this might be that such patients helped each other in medication adherence and for close follow ups. Hence, a partner may remind a patient to take pills after proper dietary instruction^30^. The result obtained in this regard is opposed by another study^31^. This result also needs further investigation.

Educated patients have better understanding about the use of dietary and medication adherence for their health status as compared to non-educated patients. Educated patients also have access of information about early diagnosis (tests of HIV) and information about how the virus transfer among individuals as compared to non-educated patients^32^. Such access of information and test of HIV, for educated patients helps to become more adherent to medication and long live within the treatment as compared to non-educated patients^23^.

Patients with mental depression and allergic for medication becomes more non-adherent to medication and defaulted from treatment with in short period of the treatment time^33^.

Underweight (malnourished) patients are more likely to be non-adherent to medication as compared to normal BMI patients. This might be due to the fact that underweight patients are faced for shortage of balanced diet and such patients who faced scarcity of balanced diet did not follow dietary instruction and the treatment may not be effective. Non-effectiveness of the treatment leads the patients to be non-adherence to medication^34^.

The level of CD4 cell count in successive visits plays a significant role for patients to be non-adherent to medication. Hence, the higher the level of CD4 cell count, the less probable of patients being non-adherence to medication and this further leads for low risk of being defaulting from treatment. The reason for this could be, patients at better status in CD4 cell count have an easy recovery of their health status and this further encourages them to be adherent to medication and close flow- ups of the treatment^21^.

Patients at working functional status are more adherent and more likely in waiting in a treatment as compared to patients who are ambulatory or bedridden. This might be due to the fact that, ambulatory patients are financially dependent and need close supervision to ensure adherence to medication. Bedridden and ambulatory patients are more likely to be affected by medication side effects and lead to be non-adherent. This result is consistent with previous research^16^.

Patients who got social support from communities living together and free from any mental depression have less probable to be non-adherent to medication and the potential reason for this might be that such patients are encouraged to take pills on time and to follow dietary instruction to be effective in the era of HAART treatment. This result is consistent with one of the previous research^25^ but contradicted with another research^1^.

Adults are less probable to be non-adherence to medication as compared to children. This might be the fact that adult patients may have survived earlier adherence barriers, and knowing the benefits of the survival, they choose to be close followers of their medication. Due to peer pressure, children are faced with situations that lead them to be non-adherence to medication compared to adults. The other reason for children to be more non-adherent as compared to adults is that they might give emphasis for playing with their friends and forgot in taking pills on time^22^. One of the studies investigated previously and contradicted in regard to this result stated that most of the time adults are so busy in managing household and may run away from home for work without pills and they may not take such pills for the whole day^28^. Adults might be more smokers and consume more alcohol as compared to children and patients may skip medications after alcohol consumption out of fear of toxic interactions between the two^31^. Hence, this needs further investigation in the future.

Patients under WHO stage4 are less likely to be non-adherence to medication as compared to other WHO stages. At enrollment stage, patients with less advanced WHO clinical staging are related to health improvement (good status of health) and positively associated to be non-adherent to medication which suggests that patients who are asymptomatic are more likely to be non-adherent. Patients at better health conditions fell healthiness and may not dis-closed their disease to people living together and need not strictly follow up their medication. Such patients may skip in taking pills whenever they are in public. On the other hand, patients with more advanced WHO stages might make them able to take the treatment properly due to more replication of the virus in their body and less recovery of their to normal conditions. This suggests that disease severity plays a role in moderating adherence behavior. The sicker the persons the more serious they take their adherence^23^, ^25^, ^35^.

## Conclusion

In current investigation, the longitudinal relationship between non-adherence to medication and time to default from treatment for patients under HAART was analyzed using separate and joint modeling approaches. Joint model revealed the common determinants of two response variables and were more reliable to infer for the study population. Joint model had less standard error as compared to separate ones.

Joint model analysis conducted in this study found that being female HIV patients, urban patients, patients living with partners, patients disclosed their disease, adult HIV patients, HIV patients with owner of cell phone, HIV patients with more advanced WHO stages, educated patients, patients at working status, normal BMI, patients with social support, baseline CD4 cell count, follow up visits and Baseline HGB were negatively associated with non-adherence to medication and had long live with in the treatment. On the other hand, patients with mental depression, patients with less advanced WHO stages, rural residents, patients not disclosed the disease, male patients, children HIV patients, ambulatory patients were positively associated with non-adherence to medication and defaulted from treatment with short time.

## Conclusion

Non-adherence to medication and defaulted from treatment are still a great challenges in HAART program. In investigating predictors of longitudinal and survival response data especially, when the two processes are correlated, valid inferences can be made through the use of a joint modeling approach. Therefore, joint model should be preferred over separate models for longitudinal and survival data analysis.

## Recommendation

Certain groups associated positively with the event of non-adherence to medication are identified and require intervention by the concerned bodies. Therefore, for such specific groups of patients which are at high risk being non-adherence to medication and defaulted from treatment should be targeted for intervention and for long live with the virus. Adherence education and awareness creation should be conducted for such patients.

## Limitation

The data in current investigation was taken in one teaching and specialized hospital, including the data in other treatment areas may reveal additional information. The data was secondary and collected by health professionals; including primary information with additional variables (patients' alcoholic and smoking status) may also provide additional information. Such gaps can be considered as limitation of current study for future investigation.

## List of abbreviations

HAART= Highly Active Anti-retroviral Therapy; PLWHA= People Living with HIV; FMOH = Federal Ministry of Health; HIV= Human Immunodeficiency Virus; AOR= Adjusted Odds Ratio; CI= Confidence Interval; GLMM= Generalized Linear Mixed effect Model; PH= proportional Hazard.

## Declaration

### Availability of data

The data used for current investigation is available with hands of corresponding author.
